# Ovarian Cancer Stem Cells Are Enriched in Side Population and Aldehyde Dehydrogenase Bright Overlapping Population

**DOI:** 10.1371/journal.pone.0068187

**Published:** 2013-08-13

**Authors:** Kazuyo Yasuda, Toshihiko Torigoe, Rena Morita, Takahumi Kuroda, Akari Takahashi, Junichi Matsuzaki, Vitaly Kochin, Hiroko Asanuma, Tadashi Hasegawa, Tsuyoshi Saito, Yoshihiko Hirohashi, Noriyuki Sato

**Affiliations:** 1 Department of Pathology, Sapporo Medical University School of Medicine, Chuo-Ku, Sapporo, Japan; 2 Department of Surgical Pathology, Sapporo Medical University School of Medicine, Chuo-Ku, Sapporo, Japan; 3 Department of Obstetrics and Gynecology, Sapporo Medical University School of Medicine, Chuo-Ku, Sapporo, Japan; Baylor College of Medicine, United States of America

## Abstract

Cancer stem-like cells (CSCs)/cancer-initiaiting cells (CICs) are defined as a small population of cancer cells that have self-renewal capacity, differentiation potential and high tumor-initiating ability. CSCs/CICs of ovarian cancer have been isolated by side population (SP) analysis, ALDEFLUOR assay and using cell surface markers. However, these approaches are not definitive markers for CSCs/CICs, and it is necessary to refine recent methods for identifying more highly purified CSCs/CICs. In this study, we analyzed SP cells and aldehyde dehydrogenese bright (ALDH^Br^) cells from ovarian cancer cells. Both SP cells and ALDH^Br^ cells exhibited higher tumor-initiating ability and higher expression level of a stem cell marker, *sex determining region Y-box 2 (SOX2)*, than those of main population (MP) cells and ALDH^Low^ cells, respectively. We analyzed an SP and ALDH^Br^ overlapping population (SP/ALDH^Br^), and the SP/ALDH^Br^ population exhibited higher tumor-initiating ability than that of SP cells or ALDH^Br^ cells, enabling initiation of tumor with as few as 10^2^ cells. Furthermore, SP/ADLH^Br^ population showed higher sphere-forming ability, cisplatin resistance, adipocyte differentiation ability and expression of *SOX2* than those of SP/ALDH^Low^, MP/ALDH^Br^ and MP/ALDH^Low^ cells. Gene knockdown of SOX2 suppressed the tumor-initiation of ovarian cancer cells. An SP/ALDH^Br^ population was detected in several gynecological cancer cells with ratios of 0.1% for HEC—1 endometrioid adenocarcinoma cells to 1% for MCAS ovary mucinous adenocarcinoma cells. Taken together, use of the SP and ALDH^Br^ overlapping population is a promising approach to isolate highly purified CSCs/CICs and SOX2 might be a novel functional marker for ovarian CSCs/CICs.

## Introduction

Cancer stem-like cells (CSCs)/cancer-initiating cells (CICs) are defined as small population of cancer cells that have the properties of high tumor initiating ability, self-renewal ability and differentiation ability [1]–[3]. Furthermore, CSCs/CICs are shown to be resistant to standard cancer therapies including chemotherapy and radiotherapy; therefore, CSCs/CICs are responsible for cancer relapse after treatment [4], [5]. Several approaches have been described to identify CSCs/CICs, including isolation by CSC/CIC-specific cell surface marker expression (e.g. CD44, CD133, CD166), detection of side population (SP) cell phenotype by Hoechst 33342 exclusion and detection of aldehyde dehydrogenase 1 (ALDH1) activity in the ALDEFLUOR assay [6]. However, the expression of cell surface markers, SP cells and the expression of ALDH1 are not related to tumor-initiating ability in some reports [7]–[9]. These observations thus suggest that these stem cell markers (cell surface markers, SP cells and ALDH1) are not functional and not necessary for maintenance of CSCs/CICs. These markers may not define high tumorigenic CSCs/CICs, and these markers are thus merely surrogate markers for CSCs/CICs. Therefore, functional non-surrogate marker which is essential for maintenance of CSCs/CICs is expected.

Ovarian cancer is one of the major malignancies and causes the death of more than one million people in the world every year [10]. In addition, most patients have miserable episodes of ascites, especially in advanced stages. To improve the clinical treatment of ovarian cancer, ovarian cancer stem cell research has emerged as a recent topic. CD44 cell surface marker, SP cells and ALDH^Br^ cells have been reported as stem cell markers for gynecological malignancies using cell lines OVCAR3, HEC-1 and other lines and primacy samples [11]–[14], and CSC/CIC research may improve the outcome of advanced ovarian cancer patients.

To improve the methods for isolation of highly purified ovarian CSCs/CICs, we analyzed the combination of known ovarian CSC/CIC markers. We analyzed ovarian cancer cell lines by SP analysis and ALDEFLUOR assay and found that SP cells and ALDH^Br^ cells were higher tumorigenic than those of main population (MP) cells and ALDH^Low^ cells, respectively. We found that the overlapping population of SP cells and ALDH^Br^ cells (SP/ALDH^Br^) were more highly tumorigenic. And we found that *SOX2* was expressed in an SP/ALDH^Br^ population at higher level, and gene knockdown of *SOX2* abrogated the tumor-initiation of ovarian cancer cells. Therefore, SOX2 might be a novel functional marker for ovarian CSCs/CICs and SP/ALDH^Br^ population is more suitable population for analysis of ovarian CSCs/CICs than SP cells or ALDH^Br^ cells.

## Materials and Methods

### Ethics Statement

Mice were maintained and experimented on in accordance with the guidelines of and after approval by the Committee of Sapporo Medical University School of Medicine, Animal Experimentation Center under permit number 08-006. Any animal found unhealthy or sick were promptly euthanized. Immunohistochemical staining study was approved by Institutional Review Boards (IRB) of Sapporo Medical University Hospital. We obtained written informed consent from all patients according to the guidelines of the Declaration of Helsinki.

### Cell lines and culture

Human ovarian cell lines (MCAS, HTBoA, OVCAR3, OVSAHO) and human endometrial carcinoma (HEC-1) cells were obtained from ATCC (Manassas, VA, USA). MCAS and HEC-1 cells were maintained in Minimun Essential medium (MEM) (Life Technologies, Grand Island, NY, USA). HTBoA and OVCAR3 cells were maintained in Dulbecco's modified Eagle's medium (DMEM) (Sigma-Aldrich, St Louis, MO, USA). OVSAHO cells were maintained in RPMI1640 medium (Sigma-Aldrich). Each cell line was supplemented with 10% FBS and cultured in a humidified 5% CO_2_ incubator at 37°C.

### Side population (SP) assay

Side population (SP) analysis was performed as described previously with some modifications [15], [16]. Hoechst 33342 (Lonza, Walkersville, MD, USA) dye was used at the concentration of 2.5 or 5.0 µg/ml in the presence or absence of verapamil (50 mM; Sigma-Aldrich) as an inhibitor of the ABC transporter. The cells were incubated at 37°C for 60 min or 90 min with continuous shaking. One million of stained cells were analyzed by FACS Aria II (BD Biosciences, San Jose, CA, USA). The Hoechst 33342 dye was excited at 357 nm and its fluorescence was analyzed using dual wave-lengths (blue, 402–446 nm; red, 650–670 nm).

### ALDEFLUOR assay

Aldehyde dehydrogenase (ALDH) activity was detected using an ALDEFLUOR assay kit (StemCell Technologies) according to the manufacturer's protocol [17]. Cells were stained by bodipy-aminoacetaldehyde (BAAA) at 1.5 mM and incubated for 30 min at 37°C. An inhibitor of ALDH1, diethylamino- benzaldehyde (DEAB), at a10-fold molar excess was used as a negative control. One million of stained cells were analyzed by FACS Aria II. The brightly fluorescent ALDH1-expressing cells (ALDH1^Br^) were detected in the green fluorescence channel (520–540 nm).

### SP and ALDEFLUOR dual staining

The cells were stained by Hoechst 33342 dye and then stained by BAAA. One million of SP and ALDEFLUOR-dual-stained cells were analyzed by FACS Aria II. The cells were divided into three groups according to ALDH intensity (ALDH^Br^ (ALDH bright), ALDH^Mid^ (ALDH middle), ALDH^Low^ (ALDH low)), then analyzed by SP assay.

### Immunohistochemical staining

Immunohistochemical staining using formalin-fixed paraffin-embedded sections of surgically resected ovarian carcinoma tissue was performed as described previously [18]. Anti-ALDH1 mouse antibody was used at 250-times dilution. Anti-ABCG2 rabbit polyclonal antibody (Sigma-Aldrich) was used at 5 µg/ml. Peroxidase-labeled goat anti-rabbit polyclonal antibody (Nichirei, Tokyo, Japan) was used as manufacturer's protocol and visualized by DAB. Alkaline phosphatase-labeled goat anti-mouse polyclonal antibody (Nichirei) was used as manufacturer's protocol and visualized by New Fuchsin (Nichirei). Membrane brown staining was judged as positive staining for ABCG2, and cytoplasm red staining was judged as positive staining for ALDH1.

### Xenograft transplantation

Sorted cells were collected and re-suspended at concentrations of 10^2^–10^4^ cells per 50 µl of PBS and then mixed with 50 µl of matrigel (BD Biosciences). The cell-matrigel mixture was injected in the subcutaneous space of 6-week-old non-obese diabetic/severe combined immune-deficiency (NOD/SCID) mice (NOD.CB17-Prdkcscid/J, Charles River Laboratory, Yokohama, Japan) under anaesthesia. Tumor growth was monitored weekly, and tumor volume was calculated by XY^2^/2 (X = long axis, Y = short axis).

### Sphere formation assay

Spherical colony formation assay was performed using CSC Complete Recombinan Medium (Cell Systems Corporation, Kirkland, WA, USA). SP/ALDH^Br^, SP/ALDH^Low^, MP/ALDH^Br^ and MP/ALDH^Low^ cells were plated at 10^3^ cells per well in 6-well ultra-low attachment plates (Corning Inc., Corning, NY, 14831) and cultured for 10 days. The morphology of the cells was assessed and pictures were taken under a light microscope every day. Round cell clusters larger than 100 µm were judged as spheres.

### Cell viability assay

For cell viability assay, SP/ALDH^Br^, SP/ALDH^Low^, MP/ALDH^Br^ and MP/ALDH^Low^ cells were isolated. Then, the cells were plated at 1000 cells per 96-well plate for 1 day and then were treated with cisplatin for 3 days under several concentrations. Subsequently, the cell viability was investigated using the Premix WST-1 Cell Proliferation Assay System (Takara Bio Inc., Otsu, Japan) according to the manufacturer's protocol.

### Adipocyte differentiation assay

For adipocyte differentiation assay, SP/ALDH^Br^, SP/ALDH^Low^, MP/ALDH^Br^ and MP/ALDH^Low^ cells were isolated. The cells were plated at 10000 cells per 24-well plate, and were grown in serum-reduced RPMI∶DMEM (1∶1) medium containing 0.5 mM trans-retinoic acid (Sigma-Aldrich) and 50 nM insulin (Sigma-Aldrich) for 2 days followed by the addition of adipose differentiation medium containing 170 nM insulin, 2 nM triiodothyronine and 0.5 mM rosiglitazone [19]. Cells were maintained in the medium for 5 days and neutral lipid accumulation was detected in 4% formaldehyde fixed cells using Oil red O (Sigma-Aldrich) staining. Lipid staining was observed using microscope, and lipid stained cells were counted.

### *SOX2* mRNA knockdown by siRNA

A *SOX2* gene knockdown experiment was performed using small interfering RNA (siRNA). SOX2 siRNA (NM003106) and negative control siRNA were purchased from Life Technologies. MCAS cells were seeded into a 24-well plate, and transfections were carried out using Lipofectamine RNAi max (Life Technologies) in Opti-MEM according to the manufacturer's instructions. Fourty-eight hours later, the cells were analyzed for expression of *SOX2*, *ALDH1A1* and *ABCG2* by RT-PCR.

### Reverse transcription polymerase chain reaction (RT-PCR) analysis

Gene knockdown of SOX2 was confirmed by RT-PCR. Isolation of RNA and RT-PCR analysis were performed as described previously [20]. The thermal cycling conditions were 94°C for 2 min, followed by 35 cycles of 15 sec at 94°C, 30 sec at 60°C, and 30 sec at 72°C. Primer pairs used for RT-PCR analysis were 5′-TGTTAGCTGATGCCGACTTG-3′ and 5′-TTCTTAGCCCGCTCAACACT-3′ for *ALDH1A1* with an expected PCR product size of 154 base pairs (bps), 5′-CACCTTATTGGCCTCAGGAA-3′ and 5′- CCTGCTTGGAAGGCTCTATG-3′ for *ABCG2* with an expected PCR product size of 206 bps, 5′-CATGATGGAGACGGAGCTGA-3′ and 5′-ACCCCGCTCGCCATGCTATT-3′ for *SOX2* with an expected PCR product size of 410 bps, 5′-GCAGTCAACAGTCGAAGAAGG-3′, and 5′-ACCACAGTCCATGCCATCAC-3′ and 5′-TCCACCACCCTGTTGCTGTA-3′ for *glyceraldehyde-3-phosphate dehydrogenase (GAPDH)* with an expected product size of 452 bps. *GAPDH* was used as an internal control.

### Quantitative real-time PCR analysis (qPCR)

Quantitative real-time PCR was performed using the ABI PRISM 7000 Sequence Detection System (Applied Biosystems, Foster City, CA) according to the manufacturer's protocol. *SOX2* (Hs01053049_s1), *ABCG2* (Hs00184979_m1), *CD44* (Hs01075861_m1), *PROM1* (Hs01009250_m1) and *ABCB1* (Hs00184500_m1) primers and probes were designed by the manufacturer (TaqMan Gene expression assays; Applied Biosystems). Thermal cycling was performed using 40 cycles of 95°C for 15 seconds followed by 60°C for 1 min. Each experiment was done in triplicate, and normalized to the *GAPDH* gene as an internal control.

## Results

### CSCs/CICs were enriched in SP cells

**Figure 1:**
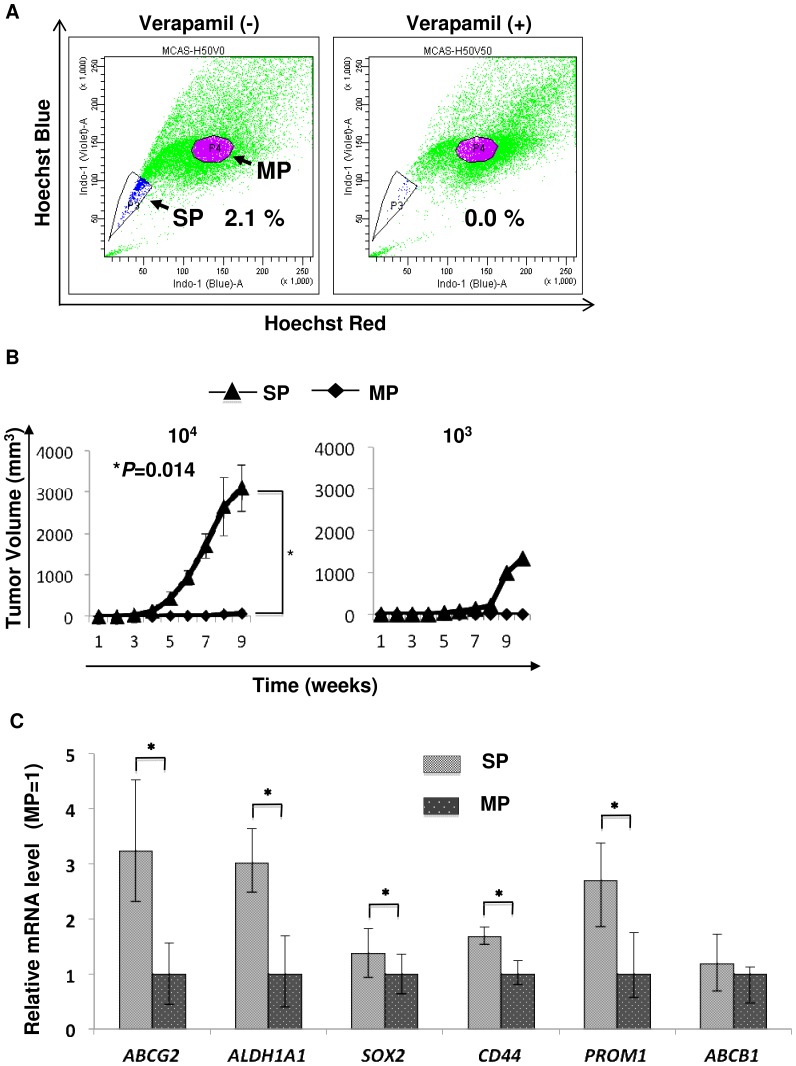
MCAS CSCs/CICs are enriched in SP cells. A. Detection of SP cells from MCAS cells. MCAS ovarian mucinous adenocarcinoma cells were stained with Hoechst 33342 dye and analyzed. The percentage represents the ratio of SP cells. B. Tumor initiation of SP cells derived from MCAS cells. 10^3^ and 10^4^ SP and MP cells derived from MCAS cells were inoculated subcutaneously into the backs of NOD/SCID mice, and tumor growth was measured weekly. Data represent means ± SD. Differences between SP and MP cells were examined for statistical significance using Student's t-test. *P values. C. qPCR of CSC/CIC markers in MCAS SP and MP cells. Data represent means ± SD. Asterisks indicate significant difference. *P<0.05. t-test.

Ovarian CSCs/CICs have been isolated as SP cells from human and mice ovarian cancer line cells [11], [21]. We analyzed several gynecological caner cell lines including human ovarian cell lines (MCAS, HTBoA, OVCAR3, OVSAHO) and human endometrial carcinoma (HEC-1) cell line (Figure 1A and Figure S1). SP cells could be detected in all line cells and the SP cell ratio were ranged from 1.2% to 2.6%. Since there is no report describing human mucinous adenocarcinoma line cell MCAS, we thus further analyzed SP cells derived from MCAS. CSCs/CICs have high tumor-initiating ability [22], we thus injected serially diluted numbers of SP cells and MP cells into the backs of three NOD/SCID mice subcutaneously to examine the tumor-initiating ability. In all three mice, tumors were initiated with 10^4^ SP cells, and tumors were initiated with 10^4^ MP cells in 2 of the 3 mice. In one mouse, a tumor was initiated with 10^3^ SP cells, while 10^3^ MP cells did not initiate any tumor (Table 1). The size of tumors derived from SP cells was significantly larger than that of tumors derived from MP cells (Figure 1B). The expression levels of stem cell markers were investigated by qPCR, and SP cells derived from MCAS cells expressed higher levels of the stem cell markers *SOX2*, *CD44* and *PROM1* and the ABC transporter gene *ABCG2*, whereas *ABCB1* was not (Figure 1C). These results indicate that CSCs/CICs were enriched in SP cells derived from MCAS cells. The results were reproduced in at least three independent experiments.

**Table 1:** Summary of tumor initiation incidence.

MCAS Cell	Tumor initiation (injected cell number)*		
	10^2^	10^3^	10^4^
SP/ALDH^Br^ cells	5/5	5/5	n.d.
SP cells	0/3	1/3	3/3
ALDH^Br^ cells	0/5	0/5	5/5
MP cells	0/3	0/3	2/3
ALDH^Low^ cells	0/5	0/5	2/5
MP/ALDH^Low^ cells	0/5	0/5	0/5

* Tumor-initiating abilities were evaluated at day 70 post cell injection. Tumor-initiation/injection.

n.d.: not determined.

### CSCs/CICs were enriched in ALDH^Br^ cells

**Figure 2:**
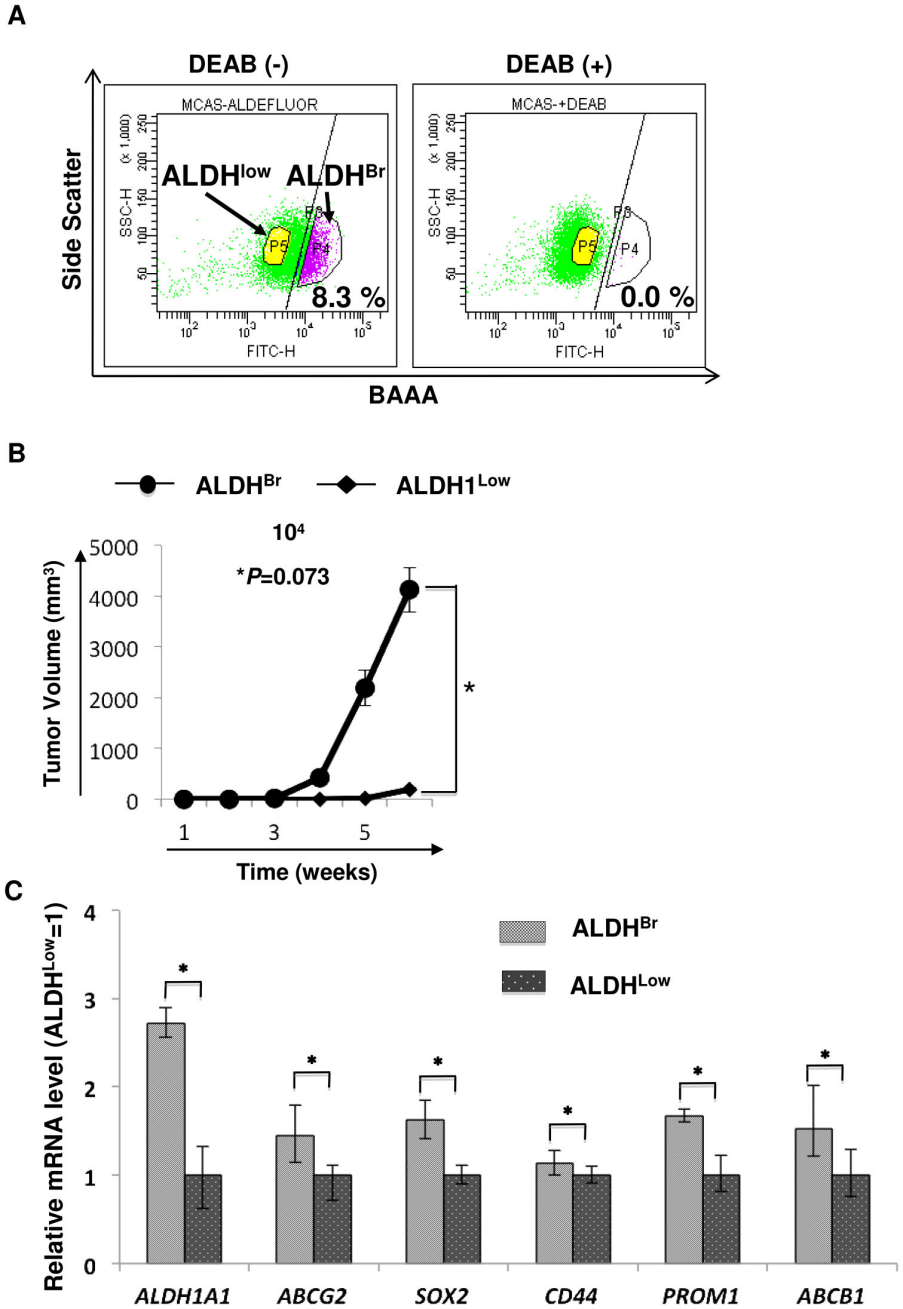
MCAS CSCs/CICs are enriched in ALDH^Br^ cells. A. Detection of ALDH^Br^ cells from MCAS cells. MCAS ovarian mucinous adenocarcinoma cells were stained with BAAA and analyzed. The percentage represents the ratio of ALDH^Br^ cells. Inhibitor indicate ALDH1 inhibitor (diethylamino- benzaldehyde (DEAB)). B. Tumor initiation of ALDH^Br^ cells derived from MCAS cells. 10^4^ ALDH^Br^ and ALDH^Low^ cells derived from MCAS cells were inoculated subcutaneously into the backs of NOD/SCID mice, and tumor growth was measured weekly. Data represent means ± SD. Differences between ALDH^Br^ and ALDH^Low^ cells were examined for statistical significance using Student's t-test. *P values. C. qPCR of CSC/CIC markers in MCAS SP and MP cells. Data represent means ± SD. Asterisks indicate significant difference. *P<0.05. t-test.

CSCs/CICs could be isolated as ALDH^Br^ cells by the ALDEFLUOR assay [23]. We therefore examined whether CSCs/CICs can be successfully isolated by the ALDEFLUOR assay. MCAS, HTBoA, OVCAR3, OVSAHO and HEC-1 cells were analyzed by the ALDEFLUOR assay and we found that the ratio of ALDH^Br^ cells was 8.1% to 11.3% (Figure 2A and Figure S1). ALDH^Br^ cells and ALDH^Low^ cells derived from MCAS were sorted and injected into the backs of five NOD/SCID mice to examine the tumor-initiating ability. In all five mice, tumors were initiated with 10^4^ ALDH^Br^ cells, while tumors were initiated with 10^4^ ALDH^Low^ cells in only 2 of the 5 mice (Table 1). The size of tumors derived from ALDH^Br^ cells was significantly larger than that of tumors derived from ALDH^Low^ cells (Figure 2B). The expression levels of stem cell markers were investigated by qPCR. ALDH^Br^ cells derived from MCAS cells expressed higher levels of the stem cell markers *SOX2*, *CD44* and *PROM1*, ALDH1A1, and the ABC transporter gene *ABCG2* and *ABCB1* (Figure 1C). These results indicate that CSCs/CICs were also enriched in ALDH^br^ cell derived from MCAS cells. The results were reproduced in at least three independent experiments.

### SP and ALDEFLUOR dual analysis

**Figure 3:**
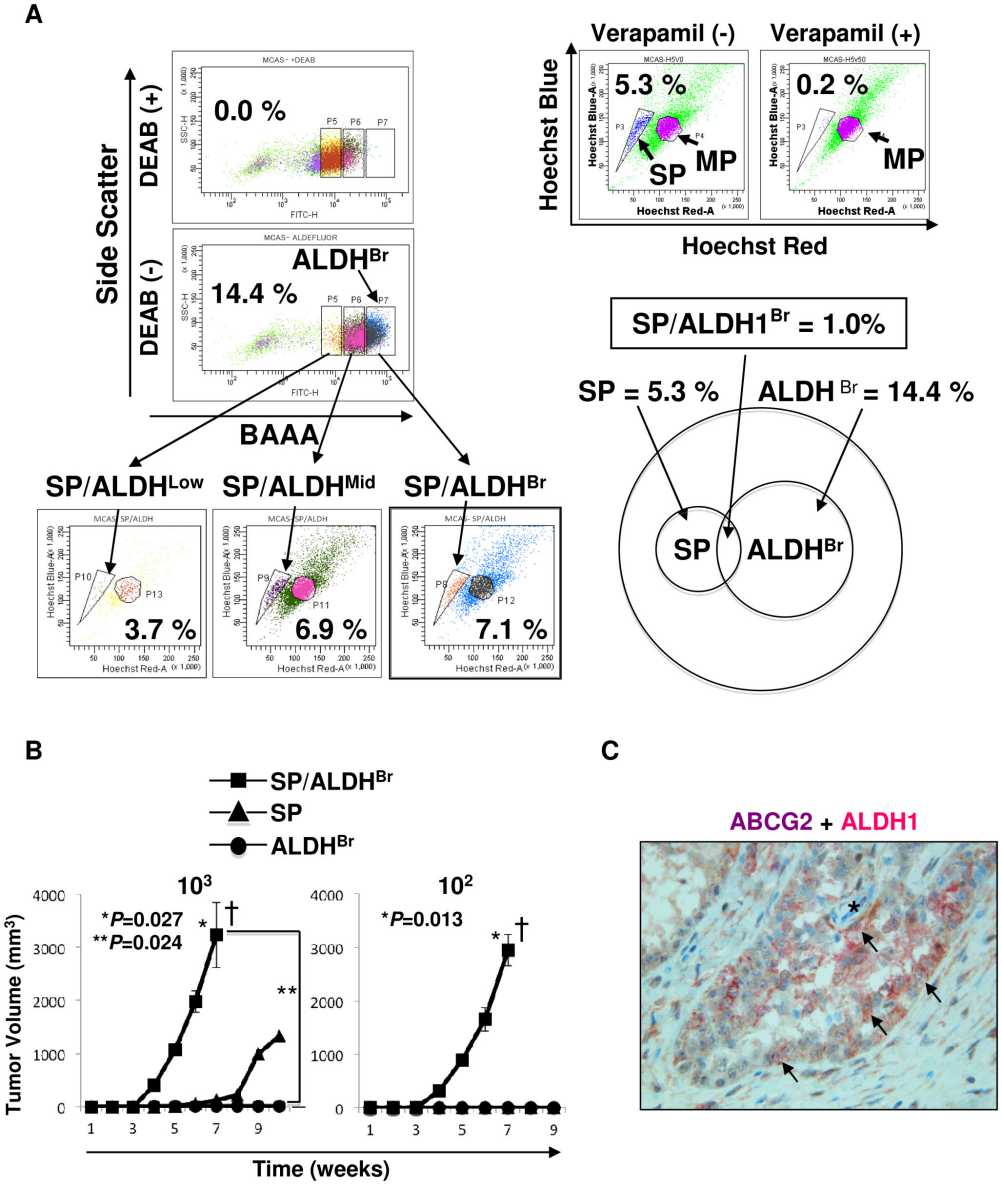
SP and ALDEFLUOR dual assay. A. Summary of SP and ALDEFLUOR dual assay. MCAS cells were stained by Hoechst 33342 dye and then stained by BAAA and analyzed. The cells were divided into three groups according to ALDH intensity (ALDH^Br^ (ALDH bright), ALDH^Mid^ (ALDH middle), ALDH^Low^ (ALDH low)), then analyzed by SP assay. The ratio of ALDH^Br^ cells was 14.4%, and the ratio of SP cells was 5.3%. The ratios of SP cells in ALDH^Br^ cells, ALDH^Mid^ cells and ALDH^Low^ cells were 7.1%, 6.9% and 3.7%, respectively. The ratio of SP/ALDH^Br^ cells in total cells was 1.0%. B. Tumor initiation of SP/ALDH^Br^, SP and ALDH^Br^ cells. 10^2^ and 10^3^ SP/ALDH^Br^, SP and ALDH^Br^ cells derived from MCAS cells were inoculated subcutaneously into the backs of NOD/SCID mice, and tumor growth was measured weekly. Data represent means ± SD. Differences between SP/ALDH^Br^ and SP cells or ALDH^Br^ cells were examined for statistical significance using Student's t-test. *P values. Daggers indicate mice death due to tumor. C. Immunohistochemical staining of ABCG2 and ALDH1. Ovarian carcinoma tissue was stained by anti-ABCG2 antibody and anti-ALDH1 antibody. Brown membrane staining indicates ABCG2 and cytoplasm pink staining indicates ALDH1. Asterisk indicates vessel, and arrows indicate ABCG2 and ALDH1 double-positive ovarian carcinoma cells. Magnification, ×400.

SP cells and ALDH^Br^ cells show greater efflux of Hoechst 33342 dye and higher expression level of aldehyde dehydrogenese, which are different phenotypes, and hematopoietic stem cells were isolated as an SP and ALDH^Br^ population in a previous study [24]. We therefore investigated the overlapping population of SP cells and ALDH^Br^ cells. After staining the MCAS cells with Hoechst 33342 dye, the cells were washed and then stained with ALDEFLUOR reagent and analyzed. In this experiment, the ratio of SP cells was 5.3% and the ratio of ALDH^Br^ cells was 14.4%. 7.1% of ALDH^Br^ cells showed SP population, and 6.9% of ALDH^Mid^ cells showed SP population, and only 3.7% of ALDH^Low^ cells showed SP population (Figure 3A). ALDH^Br^ cells exhibited partial overlapping, and only 1.0% of total cells (7.1% of 14.4% population) expressed both SP cell phenotype and ALDH^Br^ phenotype (Figure 3A).

### SP and ALDH^br^ cells have high tumor-initiating ability

The tumor-initiating ability of SP and ALDH^Br^ (SP/ALDH^Br^) cells was examined by injecting 10^3^ and 10^2^ cells, respectively, into NOD/SCID mice. Surprisingly, tumors were initiated with 10^3^ SP/ALDH^Br^ cells and with as few as 10^2^ SP/ALDH^Br^ cells in all mice (Table 1). Furthermore, tumors derived from SP/ALDH^Br^ cells showed significantly faster growth than that of tumors derived from SP cells and ALDH^Br^ cells (Figure 3B). These results indicate that SP/ALDH^Br^ cells are extremely enriched with CICs/CSCs.

We performed immunohistochemical staining to detect SP/ALDH^Br^ population in primary human ovarian carcinoma tissue. We used anti-ABCG2 antibody to detect SP cells, since SP cells are known to express higher level of ABCG2. As shown in figure 3C, dual positive (ABCG2-positive and ALDH1-positive) ovarian cancer cells were detectable in ovarian carcinoma tissue. Interestingly, some dual-positive cells exist next to vessels, might be indicating ovarian CSCs/CICs exist in vascular niche. SP/ALDH^Br^ cells were detected also from other ovarian serous adenocarcinoma line cells (OVSAHO, OVCAR3 and HTBoA) and an endometrial cell line (HEC-1), and the ratios of SP/ALDH^Br^ cells were 0.1% to 0.8% (Figure S1).

### SP/ALDH^Br^ cells have stem cell phenotypes

**Figure 4:**
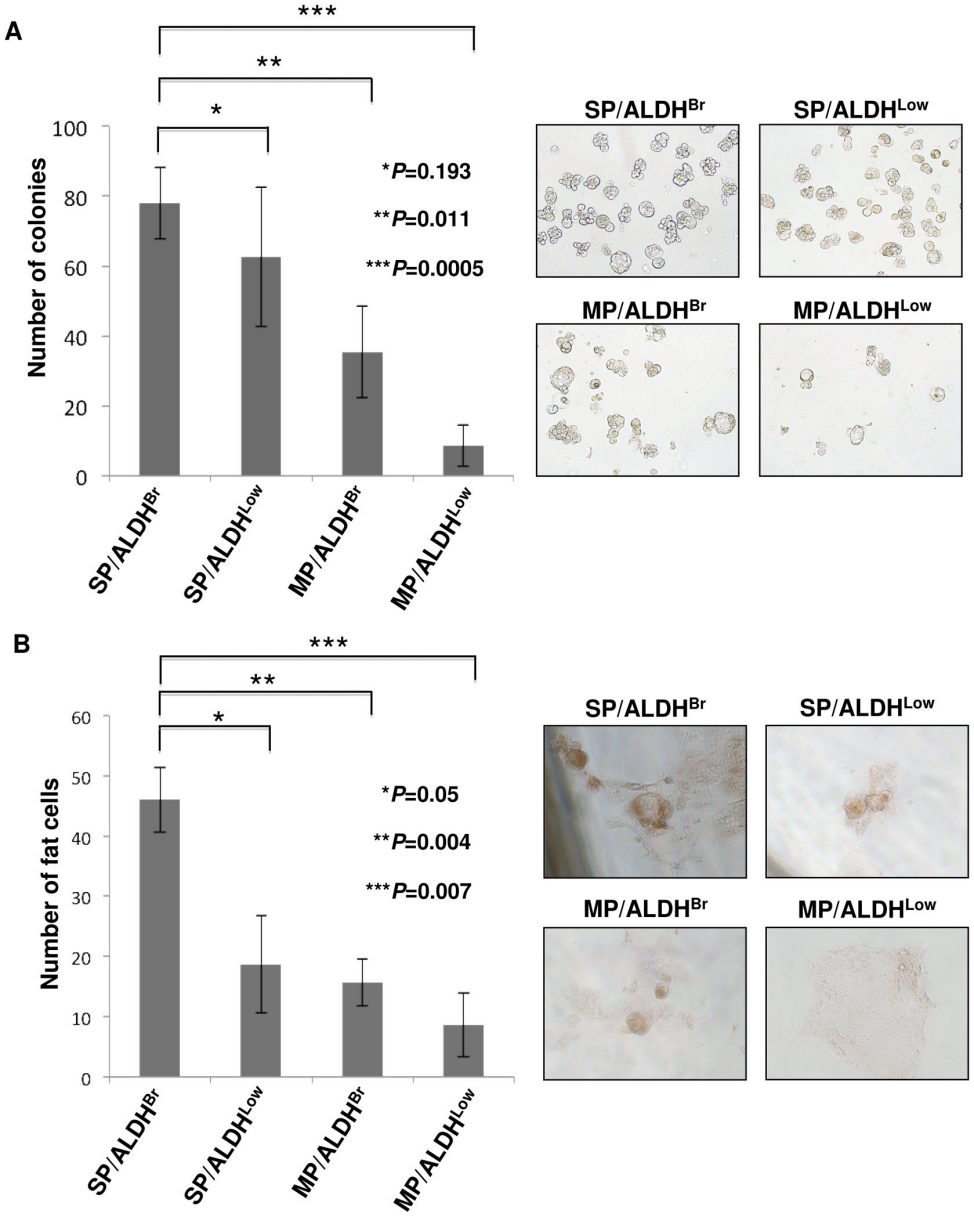
Characterization of SP/ADLH^Br^ cells. A. Sphere formation assay. The numbers of colonies from four fractions (SP/ALDH^Br^, SP/ALDH^Low^, MP/ALDH^Br^ and MP/ALDH^Low^) were evaluated at day 7. Data represent means ± SD. The differences were examined for statistical significance using Student's t-test. *P values. Representative images of spheres are shown (×100). B. Adipocyte differentiation assay. The cells were cultured under existence of trans-retinoic acid followed by adipocyte differentiation medium. Oli Red O-positive adipocytes were counted. Data represent means ± SD. The differences were examined for statistical significance using Student's t-test. *P values. Representative images of Oil Red O-staining are shown (×200). Red-staining indicate adipocyte differentiation.

The SP/ALDH^Br^ population was compared with other populations by the sphere forming assay. SP/ALDH^Br^, SP/ALDH^Low^, MP/ALDH^Br^ and MP/ALDH^Low^ cells were isolated from MCAS cells and cultured in an ultra-low attachment condition for the sphere forming assay. SP/ALDH^Br^ cells exhibited higher sphere formation efficiency than that of MP/ALDH^Br^ cells and MP/ALDH^Low^ cells (Figure 4A). The difference between SP/ALDH^Br^ cells and SP/ALDH^Low^ cells was not significant; however, SP/ALDH^Br^ cells tend to have higher sphere formation efficiency than that of SP/ALDH^Low^ cells.

CSCs/CICs have been described to have pluripotency [25], we thus analyzed the adipocyte differentiation ability of SP/ALDH^Br^ cells (Figure 4B). Isolated SP/ALDH^Br^, SP/ALDH^Low^, MPALDH^Br^ and MP/ALDH^Low^ population were cultured in an adypocyte differentiation condition. SP/ALDH^Br^ cells derived from MCAS cells revealed highest adipocyte differentiation ability compared with SP/ALDH^Low^, MP/ALDH^Br^ and MP/ALDH^Low^ cells derived from MCAS cells.

**Figure 5:**
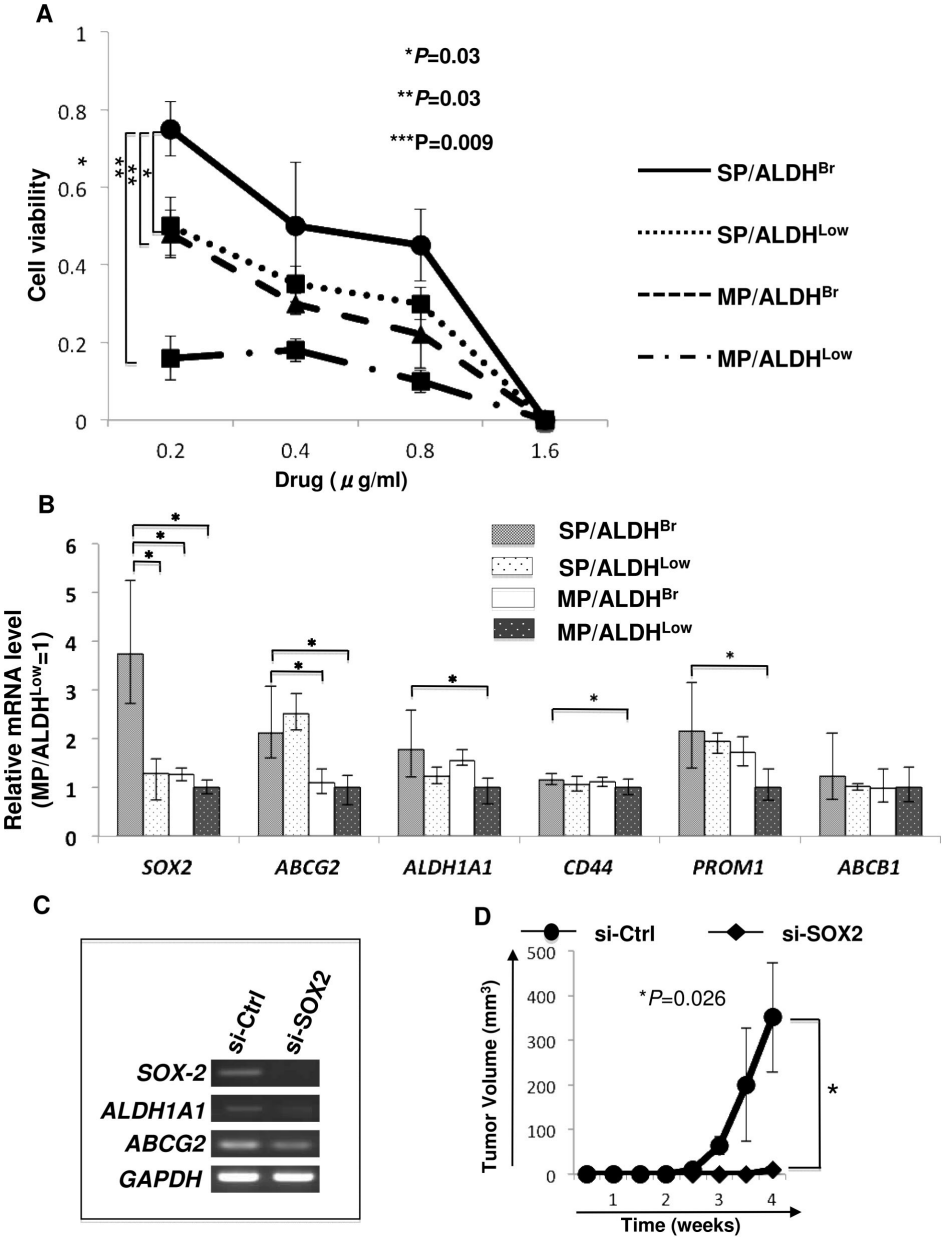
Charasterization of SP/ALDHBr cells. A. Cell viability assay. The cells were cultured under existence of serially diluted cisplatin. The viable cells were analyzed by WST-1 kit. Y-axis indicates the viability of cells. Data represent means ± SD. The differences were examined for statistical significance using Student's t-test. *P values. B. qPCR analysis. The expression of stem cell markers was examined using SP/ALDH^Br^, SP/ALDH^Low^, MP/ALDH^Br^ and MP/ALDH^Low^ cells. Data represent means ± SD. Asterisks indicate significant difference. *P<0.05. t-test. C. *SOX2* knockdown suppress the expressions of *ALDH1A1* and *ABCG2*. *SOX2* mRNA was knocked down by siRNA. Two days after transfection of SOX2 siRNA, the expressions of *ALDH1A1* and *ABCG2* were investigated by RT-PCR. *GAPDH* was used as an internal control. Control siRNA (si-Cont) transfected cells were used as negative control. D. SOX2 knock down suppress the tumor-initiation. *SOX2* mRNA was knocked down by siRNA. Ten thousand si-SOX2 and control siRNA (si-Cont) transfected cells were inoculated subcutaneously into the backs of NOD/SCID mice, and tumor growth was measured weekly. Data represent means ± SD. Differences were examined for statistical significance using Student's t-test. *P values.

CSCs/CICs are resistant to chemotherapy [4], we thus analyzed drug resistance of SP/ALDH^Br^ cells. Since cisplatin is a key drug for ovarian carcinoma chemotherapy, we used cisplatin. Isolated SP/ALDH^Br^, SP/ALDH^Low^, MPALDH^Br^ and MP/ALDH^Low^ population were cultured in an existence of cisplatin. SP/ALDH^Br^ cells significantly higher cisplatin resistance compared with SP/ALDH^Low^ cells, MP/ALDH^Br^ cells and MP/ALDH^Low^ cells. And, MP/ALDH^Low^ cells exhibited highest sensitivity to cisplatin (Figure 5A).

To analyze the molecular characteristics of SP/ALDH^Br^ population, we performed qPCR (Figure 5B). *ABCG2* mRNA was preferentially expressed in SP/ALDH^Br^ cells and SP/ALDH^Low^ cells. *ALDH1A1* was expressed in SP/ALDH^Br^ cells and MP/ALDH^Br^ cells. These expression profiles are consistent with the fact that the SP cell phenotype depends on the expression of *ABCG2* and the ALDH^Br^ cell phenotype depends on the expression of *ALDH1A1*. *SOX2* mRNA was expressed at highest level in SP/ALDH^Br^ cells but not in SP/ALDH^Low^ cells, MP/ALDH^Br^ cells or MP/ALDH^Low^ cells (Figure 5B), indicating that the SP/ALDH^Br^ population preferentially include a stem cell population. Since SOX2 has a role in the maintenance of lung CSCs/CICs [26], we investigated the relation of *SOX2* and expressions of *ABCG2* and *ALDH1A1*. The expressions of *ABCG2* and *ALDH1A1* were reduced in *SOX2* mRNA knocked down cells (Figure 5C). Furthermore, SOX2 knocked down cells showed lower tumor-initiation than that of control siRNA transfected MCAS cells (Figure 5D). Therefore, these results suggest that SP/ALDH^Br^ population express high level of stem cell gene *SOX2*, which may have role in the maintenance of CSCs/CICs and expressions of *ABCG2* and *ALDH1A1*.

**Figure 6:**
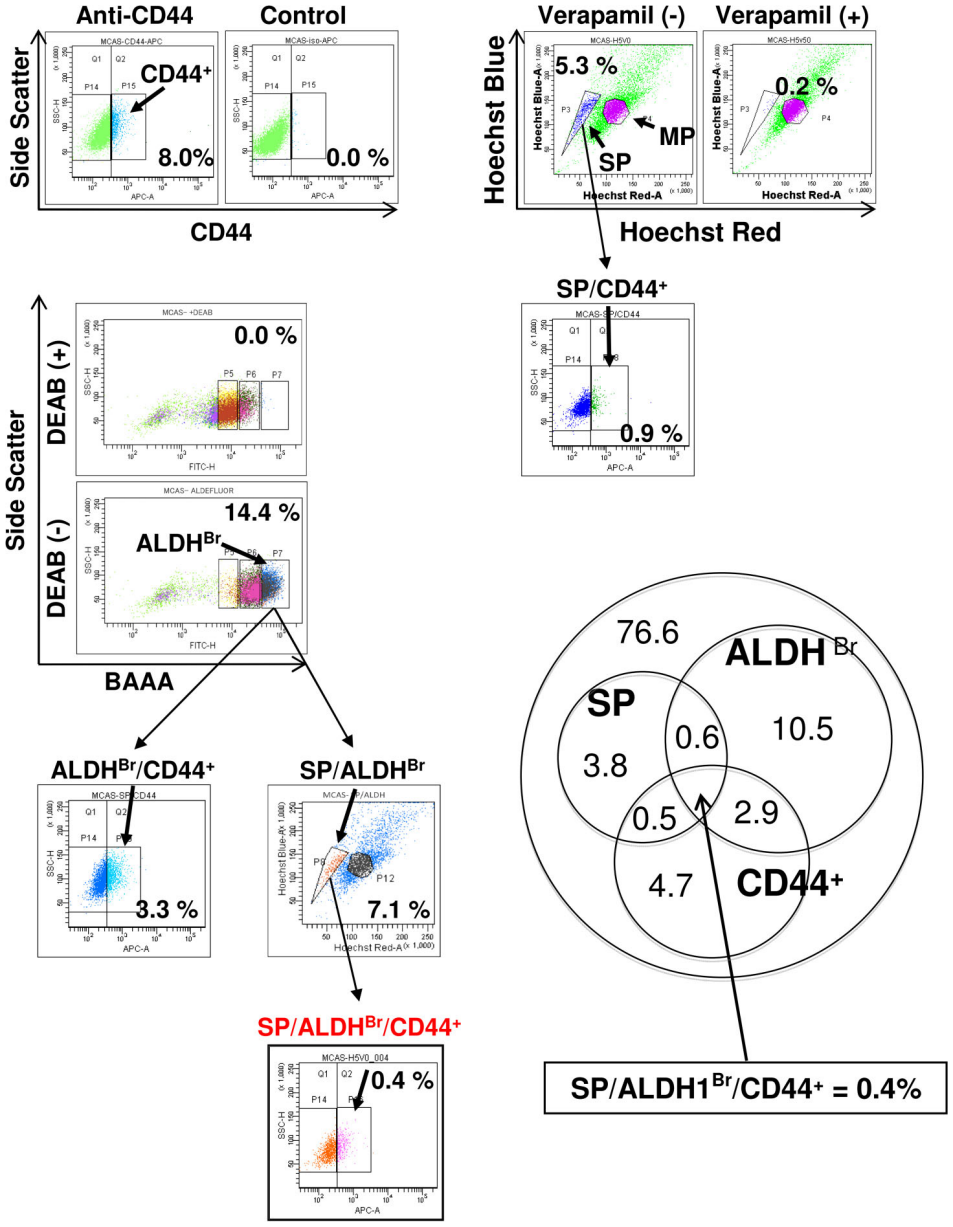
SP, ALDEFLUOR and CD44 triple staining. MCAS cells were stained by Hoechst 33342 dye, BAAA and anti-CD44 antibody, and analyzed. The ratios of SP, ALDH^Br^, CD44^+^, SP/ALDH^Br^, SP/CD44^+^, ALDH^Br^/CD44^+^ and SP/ALDH^Br^/CD44^+^ cells were 5.3%, 14.4%, 8.0%, 1.0%, 0.9%, 3.3% and 0.4%, respectively.

The expression levels of CD44, a representative marker for ovarian CSCs/CICs [27], [28], was similar in all populations (Figure 5B). We therefore investigated the relation of SP cells, ALDH^Br^ cells and CD44-positive (CD44^+^) cells. The ratio of CD44^+^ cells was 8.0%. The ratio of SP/CD44^+^ overlapping population was 0.9%, and the ratio of ALDH^Br^/CD44^+^ overlapping population was 3.3%. Furthermore, the ratio of SP/ALDH^Br^/CD44^+^ overlapping population was 0.4% (Figure 6).

## Discussion

The concept of CSCs/CICs was proposed long time ago [29]. Leukemia stem cells have been isolated from acute leukemic cells [30], [31], and the first CSC/CIC population was isolated from breast carcinoma with the combination of CD44 and CD24 expression [32]. In the following works, CSCs/CICs were successfully isolated in several malignancies. However, since the molecular mechanisms of CSCs/CICs are still elusive, accurate markers for CSCs/CICs are still unknown. Therefore, improvements in methods for isolation of CSCs/CICs are still needed.

Combination methods with double or triple markers and with markers and ALDEFLUOR assay have been reported. The populations of ALDH^Br^ and CD44^+^/CD24^−^ cells exhibited partial overlapping, and the ALDH^Br^/CD44^+^/CD24^−^ population showed higher tumor-initiating ability than that of the ALDH^Br^ population or CD44^+^/CD24^−^ population [17]. The ALDH^Br^/CD44^+^ population and ALDH^Br^/CD133^+^ population derived from human primary colon carcinoma exhibited higher tumor-initiating ability than that of ALDH^Br^, CD44^+^ and CD133^+^ populations [33]. These findings indicate that the expressions of CSC/CIC markers are partially overlapped and that the overlapped population is highly enriched with CSCs/CICs. Indeed, our results also showed similar overlapping of ALDH^Br^ cells and SP cells, and the overlapping population exhibited higher tumor-initiating ability. In an ovarian cancer study, ALDH^Br^ cells were more enriched in CD44^+^ cells than in use of the CD133^+^ cells [23], but the ALDH^Br^ and CD44^+^ overlapping was partial. We identified SP/ALDH^Br^/CD44^+^ overlapping population from MCAS cells (Figure 6). Therefore, use of the overlapping population of SP/ALDH^Br^/CD44^+^ cells may be a better approach to identify CSC/CIC populations.

Glioma stem cells have been described to differentiate into endothelial cells [34]. Malignant methotelioma stem cells have been described to differentiate into endothelial cells, neural cells and adipocytes [25]. In this study, we confirmed that SP/ALDH^Br^ cells have higher adipocyte differentiation ability. Therefore, ovarian cancer stem cells might have potential to differentiate into different lineage cells, suggesting that ovarian cancer stem cells are immature state. SOX2, a key factor for cell reprogramming [35], was expressed in SP/ALDH^Br^ cells at highest level. And knockdown of SOX2 reduced the expressions of *ABCG2* and *ALDH1A1*. Thus SOX2 might have a role to sustain immature state of ovarian cancer stem cells.

CSCs/CICs are described to be resistant to chemotherapy [4]. Indeed SP/ALDH^Br^ cells showed higher cisplatin resistance than did SP/ALDH^Low^ cells, MP/ALDH^Br^ cells and MP/ALDH^Low^ cells. Several mechanisms of drug resistance have been described, such as CSCs/CICs are dormant state, CSCs/CICs express higher levels of transporters, CSCs/CICs express higher levels of inhibitor of apoptosis proteins (IAPs) and so on. In this study, only SP/ALDH^Br^ population expressed both *ABCB1* and *ABCG2* transporters among SP/ALDH^Br^, SP/ALDH^Low^, MP/ALDH^Br^ and MP/ALDH^Low^ cells (Figure 6B). Thus, higher expressions of transporters might be one mechanisms of drug resistance of SP/ALDH^Br^ cells.

Dual SP and ALDH^Br^ cells in hematologic stem cells have been reported [24]. The overlapping population of ALDH^Br^ cells and CD133^+^ cells has prominent tumorigenicity [36]. However, SP/ALDH^Br^ cells have not been reported in solid tumors yet. SP cell phenotype represents the efflux of Hoechst 33342 dye due to the expression of ABC transporter, ABCG2, which may be involved in drug resistance [37]. ALDH^Br^ cells represent the higher expression of ALDH1, which may be involved in detoxification [38]. SP cells and ALDH^Br^ cells thus have different molecular properties, and the overlapping of SP cells and ALDH^Br^ cells were partial. And we found SP and ALDH^Br^ overlapping population was the highest CSCs/CICs enriched population which exhibited higher sphere formation, higher tumor-initiation, higher adipocyte differentiation ability, higher drug resistance and higher expression level of SOX2, a representative marker of CSCs/CICs, which is related to the tumor-initiating ability of CSCs/CICs [26]. Therefore, SP/ALDH^Br^ population is the better source of CSCs/CICs than SP cells or ALDH^Br^ cells that have been previously described. We found that SOX2 is expressed at high level in SP/ALDHBr cells, and knockdown of SOX2 suppressed the expressions of *ALDH1A1* and *ABCG2*, and suppressed tumor-initiation. Thus, SOX2 might has role in the maintenance of both SP cell and ALDH^Br^ cell population, and also has role in the maintenance of ovary CSCs/CICs.

In summary, SP/ALDH^Br^ cells comprise a more highly CSC/CIC- enriched popuration than do SP cells or ALDH^Br^ cells, and further analysis of SP/ALDH^Br^ cells should lead to elucidation of the molecular mechanisms of CSCs/CICs.

## Supporting Information

Figure S1 **SP and ALDEFLUOR dual assay.** OVCAR3, OVSAHO, HTBoA and HEC-1 cells were analyzed by SP and ALDEFLUOR dual assay. Percentages indicate the ratios of ALDH^Br^, SP and SP/ALDH^Br^ cells.
(TIF)

## References

[1] Rosen JM, Jordan CT (2009) The increasing complexity of the cancer stem cell paradigm. Science 324: 1670–1673. 19556499 10.1126/science.1171837 PMC2873047

[2] Ghaffari S (2011) Cancer, stem cells and cancer stem cells: old ideas, new developments. F1000 Med Rep 3: 23. 22162726 10.3410/M3-23 PMC3229205

[3] Hirohashi Y, Torigoe T, Inoda S, Morita R, Kochin V, et al (2012) Cytotoxic T lymphocytes: Sniping cancer stem cells. Oncoimmunology 1: 123–125. 22720232 10.4161/onci.1.1.18075 PMC3376951

[4] Dean M, Fojo T, Bates S (2005) Tumour stem cells and drug resistance. Nat Rev Cancer 5: 275–284. 15803154 10.1038/nrc1590

[5] Rich JN (2007) Cancer stem cells in radiation resistance. Cancer Res 67: 8980–8984. 17908997 10.1158/0008-5472.CAN-07-0895

[6] Tirino V, Desiderio V, Paino F, De Rosa A, Papaccio F, et al (2012) Cancer stem cells in solid tumors: an overview and new approaches for their isolation and characterization. FASEB J 10.1096/fj.12-218222 23024375

[7] Lehmann C, Jobs G, Thomas M, Burtscher H, Kubbies M (2012) Established breast cancer stem cell markers do not correlate with in vivo tumorigenicity of tumor-initiating cells. Int J Oncol 41: 1932–1942. 23042145 10.3892/ijo.2012.1654 PMC3583871

[8] Shmelkov SV, Butler JM, Hooper AT, Hormigo A, Kushner J, et al (2008) CD133 expression is not restricted to stem cells, and both CD133+ and CD133− metastatic colon cancer cells initiate tumors. J Clin Invest 118: 2111–2120. 18497886 10.1172/JCI34401 PMC2391278

[9] Burkert J, Otto WR, Wright NA (2008) Side populations of gastrointestinal cancers are not enriched in stem cells. J Pathol 214: 564–573. 18266310 10.1002/path.2307

[10] Pecorelli S, Favalli G, Zigliani L, Odicino F (2003) Cancer in women. Int J Gynaecol Obstet 82: 369–379. 14499983 10.1016/s0020-7292(03)00225-x

[11] Szotek PP, Pieretti-Vanmarcke R, Masiakos PT, Dinulescu DM, Connolly D, et al (2006) Ovarian cancer side population defines cells with stem cell-like characteristics and Mullerian Inhibiting Substance responsiveness. Proc Natl Acad Sci U S A 103: 11154–11159. 16849428 10.1073/pnas.0603672103 PMC1544057

[12] Deng S, Yang X, Lassus H, Liang S, Kaur S, et al (2010) Distinct expression levels and patterns of stem cell marker, aldehyde dehydrogenase isoform 1 (ALDH1), in human epithelial cancers. PLoS One 5: e10277. 20422001 10.1371/journal.pone.0010277 PMC2858084

[13] Zhang S, Balch C, Chan MW, Lai HC, Matei D, et al (2008) Identification and characterization of ovarian cancer-initiating cells from primary human tumors. Cancer Res 68: 4311–4320. 18519691 10.1158/0008-5472.CAN-08-0364 PMC2553722

[14] Friel AM, Sergent PA, Patnaude C, Szotek PP, Oliva E, et al (2008) Functional analyses of the cancer stem cell-like properties of human endometrial tumor initiating cells. Cell Cycle 7: 242–249. 18256549 10.4161/cc.7.2.5207

[15] Goodell MA, Brose K, Paradis G, Conner AS, Mulligan RC (1996) Isolation and functional properties of murine hematopoietic stem cells that are replicating in vivo. J Exp Med 183: 1797–1806. 8666936 10.1084/jem.183.4.1797 PMC2192511

[16] Inoda S, Hirohashi Y, Torigoe T, Morita R, Takahashi A, et al (2011) Cytotoxic T lymphocytes efficiently recognize human colon cancer stem-like cells. Am J Pathol 178: 1805–1813. 21435460 10.1016/j.ajpath.2011.01.004 PMC3078439

[17] Ginestier C, Hur MH, Charafe-Jauffret E, Monville F, Dutcher J, et al (2007) ALDH1 is a marker of normal and malignant human mammary stem cells and a predictor of poor clinical outcome. Cell Stem Cell 1: 555–567. 18371393 10.1016/j.stem.2007.08.014 PMC2423808

[18] Michifuri Y, Hirohashi Y, Torigoe T, Miyazaki A, Kobayashi J, et al (2012) High expression of ALDH1 and SOX2 diffuse staining pattern of oral squamous cell carcinomas correlates to lymph node metastasis. Pathol Int 62: 684–689. 23005595 10.1111/j.1440-1827.2012.02851.x

[19] Billon N, Iannarelli P, Monteiro MC, Glavieux-Pardanaud C, Richardson WD, et al (2007) The generation of adipocytes by the neural crest. Development 134: 2283–2292. 17507398 10.1242/dev.002642 PMC6334830

[20] Nakatsugawa M, Hirohashi Y, Torigoe T, Asanuma H, Takahashi A, et al (2009) Novel spliced form of a lens protein as a novel lung cancer antigen, Lengsin splicing variant 4. Cancer Sci 100: 1485–1493. 19459848 10.1111/j.1349-7006.2009.01187.x PMC11158687

[21] Vathipadiekal V, Saxena D, Mok SC, Hauschka PV, Ozbun L, et al (2012) Identification of a potential ovarian cancer stem cell gene expression profile from advanced stage papillary serous ovarian cancer. PLoS One 7: e29079. 22272227 10.1371/journal.pone.0029079 PMC3260150

[22] Hirohashi Y, Torigoe T, Inoda S, Takahashi A, Morita R, et al (2010) Immune response against tumor antigens expressed on human cancer stem-like cells/tumor-initiating cells. Immunotherapy 2: 201–211. 20635928 10.2217/imt.10.10

[23] Wang YC, Yo YT, Lee HY, Liao YP, Chao TK, et al (2012) ALDH1-bright epithelial ovarian cancer cells are associated with CD44 expression, drug resistance, and poor clinical outcome. Am J Pathol 180: 1159–1169. 22222226 10.1016/j.ajpath.2011.11.015

[24] Pierre-Louis O, Clay D, Brunet de la Grange P, Blazsek I, Desterke C, et al (2009) Dual SP/ALDH functionalities refine the human hematopoietic Lin-CD34+CD38− stem/progenitor cell compartment. Stem Cells 27: 2552–2562. 19650038 10.1002/stem.186

[25] Varghese S, Whipple R, Martin SS, Alexander HR (2012) Multipotent cancer stem cells derived from human malignant peritoneal mesothelioma promote tumorigenesis. PLoS One 7: e52825. 23285196 10.1371/journal.pone.0052825 PMC3532216

[26] Nakatsugawa M, Takahashi A, Hirohashi Y, Torigoe T, Inoda S, et al (2011) SOX2 is overexpressed in stem-like cells of human lung adenocarcinoma and augments the tumorigenicity. Lab Invest 91: 1796–1804. 21931300 10.1038/labinvest.2011.140

[27] Shi MF, Jiao J, Lu WG, Ye F, Ma D, et al (2010) Identification of cancer stem cell-like cells from human epithelial ovarian carcinoma cell line. Cell Mol Life Sci 67: 3915–3925. 20549538 10.1007/s00018-010-0420-9 PMC11115598

[28] Wang L, Mezencev R, Bowen NJ, Matyunina LV, McDonald JF (2012) Isolation and characterization of stem-like cells from a human ovarian cancer cell line. Mol Cell Biochem 363: 257–268. 22160925 10.1007/s11010-011-1178-6

[29] Clevers H (2011) The cancer stem cell: premises, promises and challenges. Nat Med 17: 313–319. 21386835 10.1038/nm.2304

[30] Lapidot T, Sirard C, Vormoor J, Murdoch B, Hoang T, et al (1994) A cell initiating human acute myeloid leukaemia after transplantation into SCID mice. Nature 367: 645–648. 7509044 10.1038/367645a0

[31] Bonnet D, Dick JE (1997) Human acute myeloid leukemia is organized as a hierarchy that originates from a primitive hematopoietic cell. Nat Med 3: 730–737. 9212098 10.1038/nm0797-730

[32] Al-Hajj M, Wicha MS, Benito-Hernandez A, Morrison SJ, Clarke MF (2003) Prospective identification of tumorigenic breast cancer cells. Proc Natl Acad Sci U S A 100: 3983–3988. 12629218 10.1073/pnas.0530291100 PMC153034

[33] Huang EH, Hynes MJ, Zhang T, Ginestier C, Dontu G, et al (2009) Aldehyde dehydrogenase 1 is a marker for normal and malignant human colonic stem cells (SC) and tracks SC overpopulation during colon tumorigenesis. Cancer Res 69: 3382–3389. 19336570 10.1158/0008-5472.CAN-08-4418 PMC2789401

[34] Wang R, Chadalavada K, Wilshire J, Kowalik U, Hovinga KE, et al (2010) Glioblastoma stem-like cells give rise to tumour endothelium. Nature 468: 829–833. 21102433 10.1038/nature09624

[35] Takahashi K, Yamanaka S (2006) Induction of pluripotent stem cells from mouse embryonic and adult fibroblast cultures by defined factors. Cell 126: 663–676. 16904174 10.1016/j.cell.2006.07.024

[36] Silva IA, Bai S, McLean K, Yang K, Griffith K, et al (2011) Aldehyde dehydrogenase in combination with CD133 defines angiogenic ovarian cancer stem cells that portend poor patient survival. Cancer Res 71: 3991–4001. 21498635 10.1158/0008-5472.CAN-10-3175 PMC3107359

[37] Goodell MA, Rosenzweig M, Kim H, Marks DF, DeMaria M, et al (1997) Dye efflux studies suggest that hematopoietic stem cells expressing low or undetectable levels of CD34 antigen exist in multiple species. Nat Med 3: 1337–1345. 9396603 10.1038/nm1297-1337

[38] Ma I, Allan AL (2011) The role of human aldehyde dehydrogenase in normal and cancer stem cells. Stem Cell Rev 7: 292–306. 21103958 10.1007/s12015-010-9208-4

