# Supplementary figures and images for: Ovarian Cancer Stem Cells Are Enriched in Side Population and Aldehyde Dehydrogenase Bright Overlapping Population
Source: PLoS One. 2013 Aug 13;8(8):e68187. doi: 10.1371/journal.pone.0068187 (PMC3742724; doi:10.1371/journal.pone.0068187)

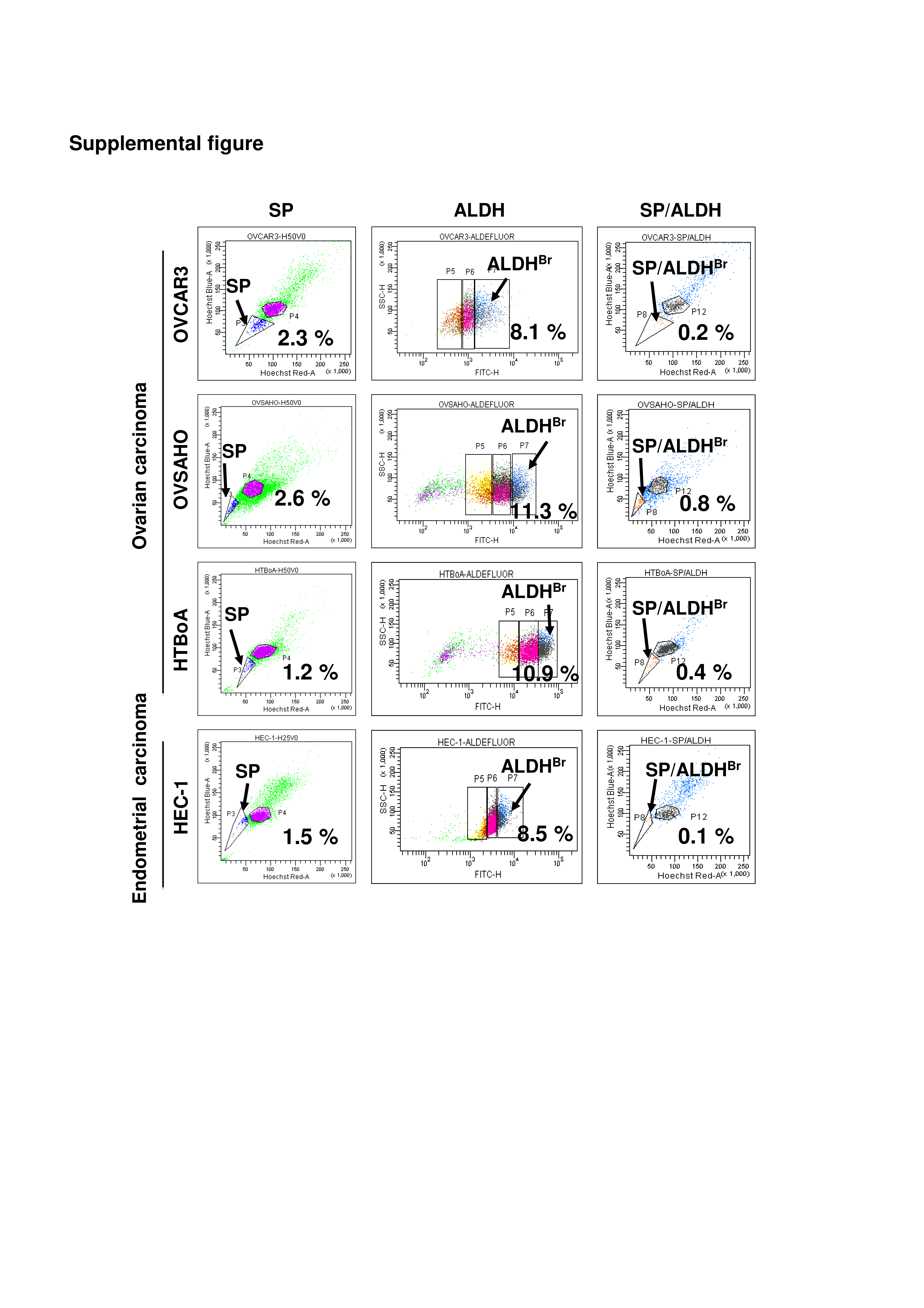

Supplement: Figure S1 — SP and ALDEFLUOR dual assay. OVCAR3, OVSAHO, HTBoA and HEC-1 cells were analyzed by SP and ALDEFLUOR dual assay. Percentages indicate the ratios of ALDHBr, SP and SP/ALDHBr cells. (TIF) [file pone.0068187.s001.tif]
